# Freezing Tolerance Enhancement and Thermographic Observation of Whole Peach Trees Applied with Cellulose Nanocrystals under Realistic Spring Frost Conditions Using a Soil–Fruit–Daylit–System

**DOI:** 10.3390/plants10112301

**Published:** 2021-10-26

**Authors:** Seongho Lee, Jae Hoon Jeong, Seung Heui Kim, Hyunsuk Shin

**Affiliations:** 1 Department of Horticultural Science, Gyeongsang National University, Jinju 52725, Korea; eucommia2@gmail.com; 2 Fruit Research Division, National Institute of Horticultural & Herbal Science, Wanju 55365, Korea; jhyskok028@korea.kr; 3 Department of Fruit Science, Korea National College of Agriculture and Fisheries, Jeonju 54874, Korea; vitis@korea.kr

**Keywords:** CNCs, frost, IR camera, peach, SFDS, whole plant

## Abstract

Due to recent abnormal weather caused by global warming, peach flowering has gradually accelerated, and spring frost damage caused by premature de-acclimation has increased. In this study, under a simulated spring frost environment using a Soil–Fruit–Daylit–System (SFDS) chamber, we investigated whether treatment with 2% cellulose nanocrystals (CNCs) could enhance the freezing tolerance of the flower buds from 2-year-old whole peach trees. Visual changes in the ice propagation were observed using an infrared camera at the same time. After the peach flower buds in the calyx red stage were placed in the SFDS chamber with a minimum temperature of −4 °C for ~20 h, the percentage of browning in the pistils and stamens was 57.0% in the control group and 14.1% in the group treated with 2% CNCs. During the first pink stage, the percentages of browning in the pistils and stamens in the control group and the group treated with 2% CNCs were 98.2% and 70.3%, respectively. However, when peach flower buds in the group treated with 2% CNCs were exposed to a −6 °C-targeted chamber, they could not mitigate frost injury. Almost all flower buds were damaged. Infrared thermal images showed that the first exotherm in the control group began at 2:33:03 am, whereas that of the group treated with 2% CNCs began at 3:01:33 am. The control started to express exothermic behavior at −4.2 °C, while the group treated with 2% CNCs started expressing exothermic behavior at −5.1 °C. Thus, treatment with 2% CNCs enhanced the freezing tolerance by −0.9 °C and delayed the first instance of exothermic behavior by ~28 min. These results indicate that treatment with 2% CNCs could mitigate the frost damage of peach flower buds in a frost environment of −5 °C.

## 1. Introduction

The average temperatures in temperate climates have risen due to global warming. Frost damage in spring is also increasing. Frost damages to buds, flowers, and developing fruits after dormancy are usually more important than those due to low winter temperatures. Peaches are a major crop among temperate fruits. Its blooming date has advanced by 11.1 days during the last 30 years [1]. This implies that peaches might be exposed to spring frost damage for a longer time.

Methods to prevent frost damage in fruit trees can be classified as active and passive. Active methods can be applied before or during frost. For example, wind machines, heaters, and sprinklers can help keep the surrounding temperatures from dropping below freezing [2,3,4]. However, these methods are expensive. In addition, they might cause noise and environmental pollution. In particular, heaters can bring about a conflagration in orchards. Passive methods are applied before frost damage occurs. These include orchard site selection, a choice of suitable cultivars, covering plants, and the application of chemical products to prevent frost damage [5]. Among them, chemical products are less expensive than active methods. In addition, they have no space limitations for treatment. However, scientific information about the chemical products that have been developed is scarce or inconclusive [6].

Cellulose nanocrystals (CNCs) are known as nano-biomaterials with low thermal conductivity and biodegradable properties. Since their porosity and stability are higher than those of other natural biomaterials, they have been widely used as materials in other fields that use their thermal insulation properties [7]. CNCs are effective against frost damage in cherries and grapes [8]. Likewise, a positive effect has been observed for crops with flower buds with a cluster structure. However, studies on their applications in other crops are scarce. Therefore, this study applied CNCs to peach trees to obtain a chemical cover effect using the insulating properties of CNCs.

Many studies have been conducted to understand the mechanisms of frost injury through controlled chamber experiments. However, studies have been conducted mainly on herbaceous plants because woody plants have relatively larger volumes to adapt to a general growth chamber than herbaceous plants. Thus, most woody plants have been used for indoor chamber experiments. The Soil–Fruit–Daylit–System (SFDS) chambers used in this study could accommodate whole fruit trees and more individuals than previously developed Soil–Plant–Atmosphere–Research (SPAR) chambers. SFDS can control the temperature, relative humidity, and concentration of CO_2_ in the chamber better than general chambers due to more detailed programming. Furthermore, as their chamber walls are made of Plexiglas, the samples placed inside can receive more realistic seasonal light intensity and quality than closed artificial chambers. Therefore, we used SFDS to maximally mimic realistic frost damage in field conditions in this experiment.

Several methods are available to assess freezing tolerance in plants, including the triphenyl tetrazolium chloride test, differential thermal analysis, and electrolyte leakage analysis [9,10,11]. Recently, infrared (IR) thermography analysis using an IR recording camera has been developed. It is a nondestructive inspection method that can measure the surface temperature of an object. This approach is based on measuring the latent heat of water inside the plant tissue [12]. IR thermography also provides detailed real-time images of the surface temperature of plant organs and allows for ice nucleation and propagation to be observed [13].

Thus, the aim of this study was to investigate whether treatment with CNCs could enhance the freezing tolerance of whole potted peach trees. Here, SFDS chambers were used to simulate a spring frost environment more realistically. An IR thermal imaging camera was used to observe frost damage more accurately without excising plants. Our results provide beneficial information to understand the spring frost mechanisms.

## 2. Results

### 2.1. Microscopic Observation of Frost Damage to Peach Flower Buds after Treatment with 2% CNCs

Damage to the stamens and pistils of peach flower buds after a spring frost event simulated with two target temperature conditions (−4 °C and −6 °C chambers) was observed using a stereoscopic microscope. Their damage rates were investigated and divided into two developmental stages (fourth (calyx red) and fifth (first pink) stages) (Table 1). For flower buds of the calyx red stage staying in the −4 °C chamber, 43% of the control showed no damage, whereas 85.9% of the flower buds in the group treated with 2% CNCs were not damaged. Thus, treatment with 2% CNCs induced significant enhancement of the freezing tolerance. The percentage of flower buds showing only pistil damage was 39.5% in the control and 1.3% in the group treated with 2% CNCs. The percentage of flower buds showing only stamen damage was 17.5% in the control and 12.8% in the group treated with 2% CNCs. Flower buds showing both pistil and stamen damage were not observed. At the first pink stage, the percentage of flower buds showing no damage was 1.8% in the control and 29.7% in the group treated with 2% CNCs. Therefore, treatment with 2% CNCs was proven to have a freeze-prevention effect at the first pink stage. Notably, the percentage of flower buds showing pistil damage was 60.2% in the control and 0.0% in the group treated with 2% CNCs. The percentage of flower buds showing only stamen damage was 70.3% in the control. On the other hand, the damage rate of flower buds in the −6 °C chamber condition was much higher than that under the −4 °C environment regardless of treatment. In particular, the control and the group treated with 2% CNCs showed frost damage rates of more than 90% irrespective of phenological stages. Although treatment with 2% CNCs numerically reduced frost damage compared with the control, the freeze-mitigating effect of 2% CNCs was somewhat scant below the −6 °C condition.

### 2.2. Thermal Image Analysis of Frost Damage to Peach Flower Buds after Treatment with 2% CNCs

**Figure 1:**
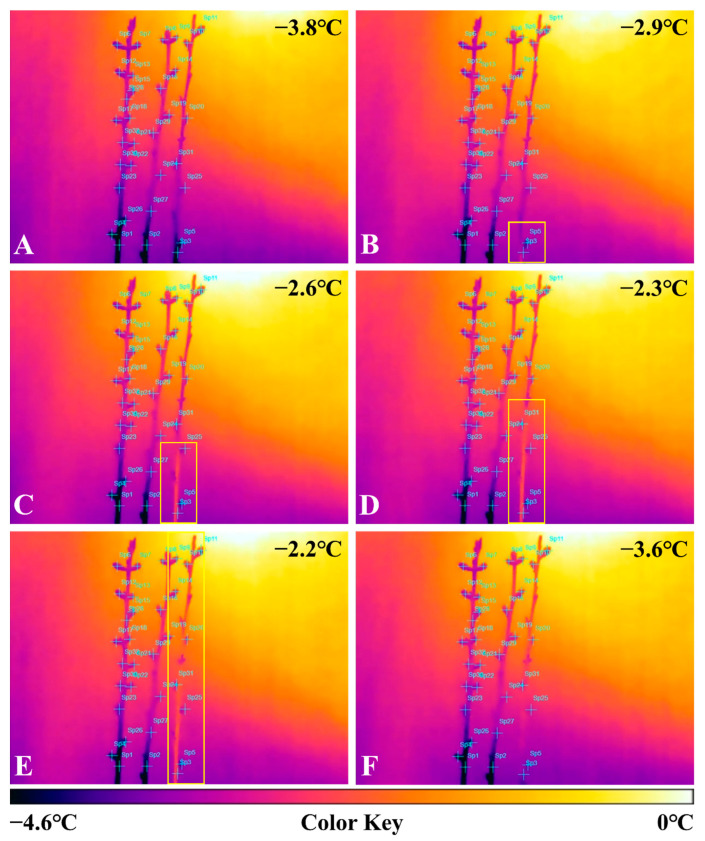
Visuals of the thermal changes in excised ‘Daewol’ peach shoots (**A**–**F**) suffering frost damage from 1 to −4 °C for 4 h. In each step, each event after expressing exothermic behavior occurred at about 20 s (**B**–**E**). Finally, about 15 min elapsed for the temperature to drop to −3.6 °C (**F**).

In the preliminary experiment, no latent heat was found for peach shoots treated with 2% CNCs (Figure 1A–F). However, the control shoots first started heating at −3.8 °C, starting from the basal part and proceeding to the apical part (Figure 1A-E). It took 80 s to heat up to the apical part. The temperature increased by about 1.6 °C. After about 15 min passed, the temperature reached −3.6 °C again (Figure 1F).

**Figure 2:**
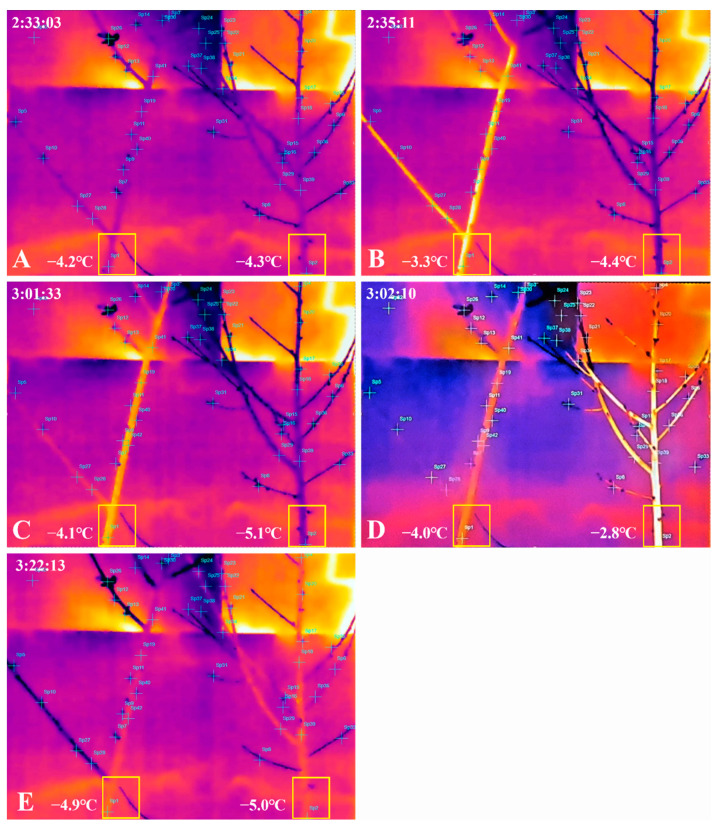
Visuals of the thermal changes in 2-year-old whole peach trees (**A**–**E**) caused by a spring frost simulation using an SFDS chamber (target temperature: −6 °C). For the temperature and relative humidity conditions, refer to Figure 3. The control is on the left, and the 2% CNCs-treated sample is on the right. The whole plant took about 2 min to radiate heat. Exothermic behavior was observed first in the control (**A**,**B**). After about 30 min, the 2% CNCs-treated plant started expressing exothermic behaviors. The whole plant took about 40 s to radiate heat (**C**,**D**). Finally, the 2% CNCs-treated plant spent about 20 min to return to −5.0 °C (E).

In the process of simulating spring frost, when water within the plant tissues showed ice nucleation under subzero temperature conditions, the latent heat generated within the plant tissues was released and detected with an IR thermal imaging camera. Each representative sample of the control and the group treated with 2% CNCs was recorded with an IR camera. The surface temperatures and ice propagation images of samples were then observed. The colors of the thermal images were close to white when the temperature went up. They were close to black when the temperature went down. The observations of major temperature changes were based on the targets in the yellow box of Figure 2.

Recording of the thermal images began at 1:22 am on April 9. No exothermic image was observed in the control and in the group treated with 2% CNCs until −4.2 °C at 2:33:03 am (Figure 2A). After that, in the control, exothermic images of the whole plant were observed at 2:35:11 am and the temperature increased from −4.2 to −3.3 °C (by ~0.9 °C) (Figure 2B). Interestingly, while it took about 2 min and 8 s for the whole plant in the control to become completely exothermic, the control spent approximately 26 min to reach the same temperature at a pre-exothermic state. On the other hand, the plant in the group treated with 2% CNCs did not show any exothermal behavior until 3:01:33 am (Figure 2C). Since then, noticeably, the whole plant in the group treated with 2% CNCs was entirely exothermic in about 37 s at 3:02:10 am. The temperature of the 2% CNCs-treated plant increased from −5.1 to −2.8 °C (by ~2.3 °C) (Figure 2D). It took approximately 20 min for the 2% CNCs-treated plant to reach the same temperature at the pre-exothermic state (Figure 2E).

## 3. Discussion

**Figure 3:**
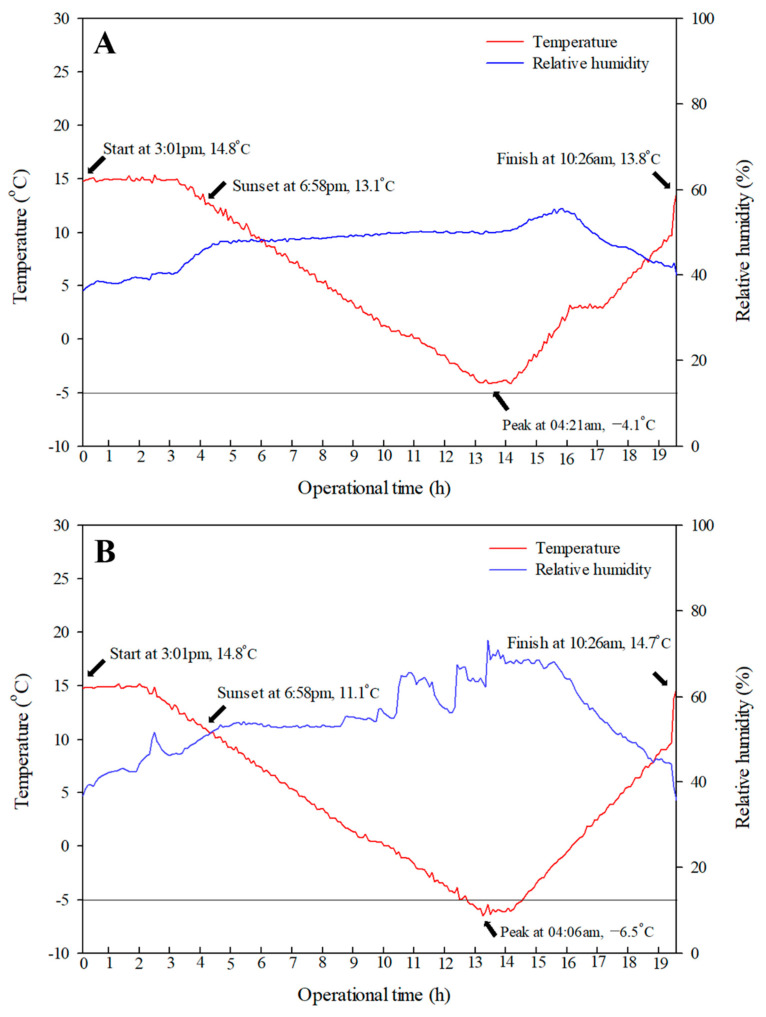
Changes in the temperature and relative humidity inside SFDS chambers set at the minimum target temperatures (−4 °C (**A**) or −6 °C (**B**)).

The risk of frost damage and resultant economic losses are steadily increasing. A remedy to effectively alleviate frost damage should be sought out. In this study, SFDS chambers (target temperatures of −4 °C or −6 °C) were used to investigate whether frost damage could be alleviated by treatment with 2% CNCs to protect peach flower buds in a controlled environment in which frost emergence was simulated (Figure 2 and Figure 3). The differences in frost damage and ice propagation between the control group and the group treated with 2% CNCs were observed using a stereoscopic microscope and an IR camera.

In the −4 °C chamber, the control showed significant frost damages due to a frost-simulated environment. Fully open flowers generally can withstand only −1 °C to −3 °C of frost [14]. This means that the SFDS chamber had well-established frost damage occurring in spring field conditions. The more the phenological stage of the ‘Daewol’ peach flower buds progressed, the more the control showed severe frost damages (Table 1). Simons and Doll [15] reported that frost damage highly depends on the developmental stage of flower buds. On the other hand, flower buds in the group treated with 2% CNCs showed a decrease in frost damage in the −4 °C chamber (Table 1). In the calyx red stage, the damage rate of flower buds in the group treated with 2% CNCs decreased by 42.9% compared with that in the control. In the first pink stage, it decreased by 27.9% in the group treated with 2% CNCs compared with that in the control. The film of 2% CNCs has a thermal conductivity of 0.061 W m^−1^ K^−1^. It has a lower thermal conductivity than polypropylene insulation or polyethylene terephthalate developed for insulation [8]. For this reason, treatment with 2% CNCs is thought to be able to reduce the damage rate of flower buds by acting as an insulation material that increases resistance to changes in external temperatures caused by frost. These results were similar to previous results showing an increase of 2–4 °C in freezing tolerance on the flower buds of grapes and cherries treated with 2% CNCs [8]. However, previous studies on frost damage to peach flower buds did not deal with damage to stamens and pistils separately in detail. Interestingly, in the −4 °C chamber, the damage rate of pistils of flower buds treated with 2% CNCs was significantly reduced at both the calyx red and first pink stages compared with that of the control. In the calyx red stage, the damage rate of pistils was 39.5% in the control and 1.3% in the group treated with 2% CNCs. At the first pink stage, the damage rate of pistils was 60.2% in the control and 0% in the group treated with 2% CNCs (Table 1). The damage rate of stamens in the group treated with 2% CNCs decreased by 4.7% in the calyx red stage compared with that in the control group. However, the difference in damage rate between the two groups was insignificant in the first pink stage. Spring frost can dramatically affect the browning of flower buds and fruit sets [16]. Through this experiment, the treatment with 2% CNCs was found to be able to reduce the damage of pistils caused by spring frost significantly. In addition, only stamen-damaged flowers are thought to improve the defect of fruit sets through artificial pollination. In the −6 °C chamber, the treatment with 2% CNCs was found to reduce the damage rate of flower buds compared with the control, although such an effect was somewhat marginal (Table 1). Therefore, the treatment with 2% CNCs was considered to not effectively alleviate frost damage at a temperature less than −6 °C.

Freezing patterns using IR cameras in low-temperature environments have been observed for several plants [17,18,19,20]. In this preliminary experiment, the exact temperature rise, and the time taken to generate heat on a peach tree’s excised shoots during the freezing process have not yet been revealed. However, our preliminary experiment showed a pattern of specific ice propagation on peach shoots without bacteria inoculation. Wisniewski et al. (1997) reported that the spread of ice nuclei begins from inoculated areas when ice nucleation bacteria are inoculated on the excised shoots of apples or peaches. If no bacteria are inoculated, the spread of the ice nuclei occurs in the stems.

Previously, experiments observing whole woody plants with IR cameras in a frost environment have not been reported. In addition, the freezing patterns of whole peach trees have not yet been reported. We observed that ice nuclei in whole peach trees occur at −4.2 °C in the −6 °C chamber (Figure 2B). The ice nuclei were first observed in the trunk. The transmission of ice nuclei proceeded from the basal part to the side shoots and the apical part. These results are similar to the freezing pattern observed for wheat leaves in an external low-temperature environment of about −8 °C and a controlled environment but differed from the sequential pattern of freezing from old to new leaves (Livingston et al., 2018). Even in blackberry shrub plants, the ice nuclei were first observed in the stem under controlled conditions reaching −4 °C for 15 h. The ice nuclei then spread to the flower buds and blooming flowers [19]. However, since blackberry plants lay flower shoots down for six weeks to grow upward, the freezing pattern of a typical blackberry plant may differ from our experimental results. The propagation rate of ice nuclei also differed depending on the type of crop and experimental conditions. The leaves of wheat took about 15 s for the ice nuclei to spread, and the stem of a blackberry took 20 s. The propagation of the ice nuclei of peach excised shoots took 80 s, while the whole plant took about 2 min. In conclusion, the type of plant, the volume, and the presence or absence of woody parts of a plant can affect the propagation speed of ice nuclei.

Additionally, treatment with 2% CNCs resulted in different propagation temperatures and times of ice nuclei formation compared with the control. In the control, heat generation first started at −4.2 °C, and the temperature rose to −3.3 °C, whereas after treatment with 2% CNCs, heat generation started at −5.1 °C and the temperature rose to −2.8 °C. Notably, the control took about 2 min and 8 s for the ice nuclei to propagate entirely to the apical part. However, peach trees treated with 2% CNCs radiated heat within 37 s. This fact means that ice nuclei that formed at lower temperatures could propagate faster. The control took 28 min to return to the temperature before exothermic activity, but the group treated with 2% CNCs took only 20 min. This result was different from blackberry plants, which took 52 min to return to the temperature before exothermic activity (Takeda and Glenn, 2016). In this experiment, an increase in temperature in whole peach trees meant that latent heat was generated when an ice nucleus was formed. Therefore, the temperature and time at which latent heat was first observed meant the temperature and time at which the ice nucleus first occurred. Therefore, treatment with 2% CNCs could lower the temperature at which ice nuclei occur in peach trees and could delay the time until dehydration damage is caused by the ice nuclei. These results are similar to results showing that plants treated with hydrophobic kaolin films on tomato leaves have a freezing delay while the control is damaged at −6.0 °C [21].

## 4. Materials and Methods

### 4.1. Plant Material and Treatment with CNCs

Two-year-old ‘Daewol’ peach trees commercially grafted on wild peach seedlings were planted in rubber pots (540 × 500 mm in diameter and height). The trees were used in this experiment after the chilling requirement was met in a natural condition. The CNCs solution was prepared by dispersing cellulose nanocrystals spray-dried powders in deionized water. The control (deionized water) and treatment with 2% CNCs were applied using a compressed sprayer until each solution ran off the trees on 7 April 2021. Both treatments were dried completely in the field for more than 12 h to prevent freezing before putting the trees into the SFDS chamber. Six trees were used for each treatment.

### 4.2. Observation of 2% CNCs-Treated Excised Shoots Using an IR Camera

Prior to the simulations of spring frost, we first observed the freezing process of excised ‘Daewol’ peach shoots as a preliminary experiment. On 24 March, three excised peach shoots were collected from the 2-year-old shoots of 10-year-old ‘Daewol’ peach trees and placed on a floral foam at 15 °C. On the next day, the 2% CNCs solutions were sprayed onto 40 cm excised shoots (in Figure 1, left and center). The control was sprayed with deionized water (in Figure 1, right). These shoots were then placed on the floral foam at 15 °C for 24 h. Thereafter, they were placed in a low-temperature chamber that decreased from 10 °C by 2 °C per h. Finally, the target temperature was set to reach −4 °C. They were then observed with an IR camera.

### 4.3. Simulation of Spring Frost Using SFDS Chambers

**Figure 4:**
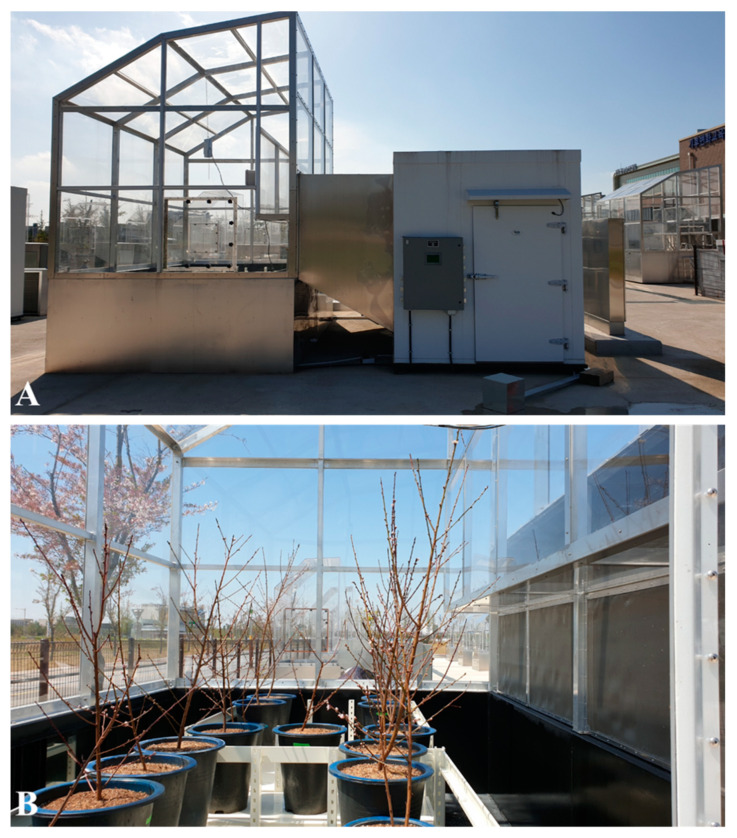
A front view of an SFDS chamber (**A**) and 2-year-old ‘Daewol’ peach trees placed in the SFDS chamber (**B**).

The simulation of spring frost was conducted using two units of SFDS (Environmental Growth Chamber, Chagrin Falls, OH, USA) chambers located at the Korea National College of Agriculture and Fisheries, Wanju, Korea. Each SFDS chamber was 3256 × 4913 × 3581 mm (length × width × height) in size and was covered with Plexiglas (Figure 4A). On April 8, the control and the group treated with 2% CNCs were placed in each chamber (Figure 4B). The lowest temperature of one unit was set at −4 °C (Figure 3A), and the other unit was set at −6 °C (Figure 3B). The operational time of both units lasted about 19 h. As shown in Figure 3A,B, the simulation of each chamber began at 3:01 pm. The targeted chambers at −4 °C and −6 °C started to operate from 14.9 °C at 6:06 pm and from 15.0 °C at 5:01 pm, respectively. The temperatures of both chambers dropped at rates from 1 to 2 °C per h until 4:00 am on April 9. After sunset at 6:58 pm, the temperature inside the chamber continued to drop to each lowest set temperature without being affected by sunlight. Similar to natural frost damage, the lowest temperature was maintained for 1 h from 4:00 to 5:00 am on April 9. After that, the temperature of each chamber was increased gradually by 3 °C per h. The relative humidity was set at 70%, and the CO_2_ concentration was set at 300 ppm during the operating time.

### 4.4. Evaluation of Frost Damage of Peach Flower Buds

**Figure 5:**
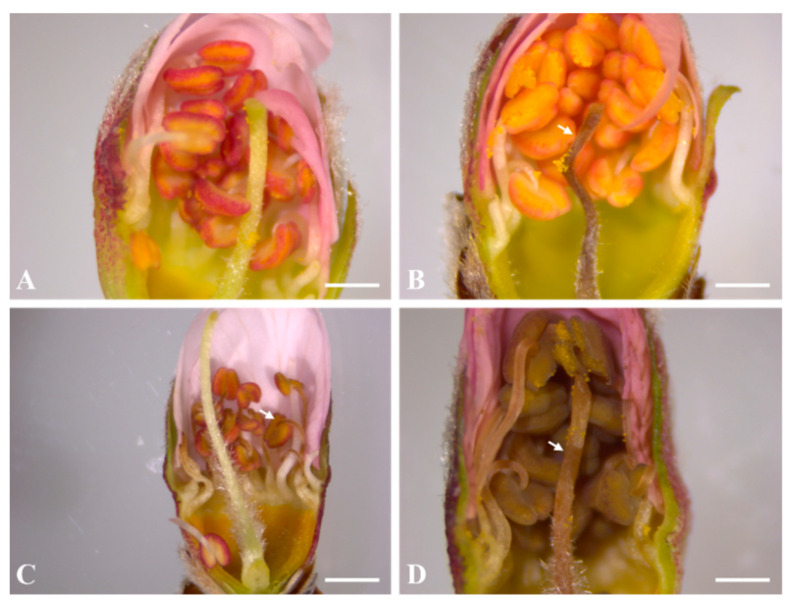
Representative visuals of damage to the peach flower buds caused by spring frost simulation using an SFDS chamber. Browning of each reproductive organ was evaluated by examining the transverse section of a peach flower bud (**A**–**D**). (**A**), no damaged; (**B**), only pistil damaged; (**C**), only stamen damaged; and (**D**), pistil + stamen damage. The scale bars are 500 µm in (**A**,**B**,**D**) and 400 µm in (**C**).

After simulating spring frost, the peach flower buds on the sylleptic shoots of two-year-old ‘Daewol’ peach trees were classified by the fourth (calyx red) and fifth (first pink) stages based on the general phenological development of the peach flower buds. Sampling was performed at the same time by taking buds with different degrees of development. At least 17 flower buds were evaluated per tree, and at least 6 flower buds were evaluated per stage. The fourth stage was from the calyx red stage to 50% emergence of the whole petals, and the fifth stage was from the first pink bloom to the pre-blooming stage. After dissecting the flower buds, the browning reproductive organs were observed using a stereoscopic microscope (stemi305, Zeiss, Germany). Damage to the flower buds was classified as follows: no damage, only pistil damage, only stamen damage, or pistil + stamen damage (Figure 5). No damage was assessed as the reproductive organs not having browned (Figure 5A). Browning of the stigma and/or style was considered pistil damage (Figure 5B). In addition, browning of more than one stamen (the anther and/or the filament) was considered stamen damage (Figure 5C). Finally, browning of both reproductive organs was considered pistil + stamen damage (Figure 5D). The number of flower buds damaged at each stage was expressed as a percentage: number of flower buds with damaged organs/number of all flower buds per developmental stage × 100.

### 4.5. Observation of Frost Events Using an IR Camera

Ice nucleation and propagation of the control and 2% CNCs-treated peach trees suffering from spring frost simulation using SFDS chambers were observed using an IR camera (E75, FLIR Systems, North Billerica, MA, USA). One of the six trees in each treatment was selected as a representative whole peach tree. The representative whole peach trees of each treatment were placed side by side. An IR camera with a tripod was installed 1 m away from the representative whole peach trees. It was connected to a laptop in the −6 °C-targeted chamber. The IR camera emissivity was set at 0.97. Non-reflective paper was installed behind the samples to prevent radiant heat from being reflected by the Plexiglas while the IR camera was recording. Recording of the infra-thermography connected to a laptop began when the inside temperature of the chamber reached 0 °C. It finished when the battery of the IR camera ran out in about three hours. About 40 points of thermal sites on the stems and flower buds were selected from the whole plant. The thermography images were analyzed using FLIR Tools (FLIR Systems, North Billerica, MA, USA).

## 5. Conclusions

Due to abnormal weather caused by global warming, frost damage is expected to increase during the flowering season of peaches. We investigated whether treatment with 2% CNCs could alleviate the frost damage of peach flower buds in a frost-simulated environment using SFDS chambers. We conclude that 2% CNCs acted as an insulating material that reduces frost damage to buds. Differences in the frost damage and ice propagation between the control group and the group treated with 2% CNCs were observed using the stereoscopic microscope and an IR camera. Treatment with 2% CNCs showed a significant reduction in pistil damage, the ice nucleation temperature was lowered by −0.9 °C compared with the control, and its ice nuclei occurred at −5.1 °C. Our results suggest that treatment with 2% CNCs can mitigate frost damage to peach flower buds at temperatures higher than −5 °C.

## Figures and Tables

**Table 1:** Damage^z^ to the flower buds of 2-year-old potted ‘Daewol’ peach trees caused by spring frost simulation (target temperatures: −4 °C or −6 °C) in SFDS chambers.

Set Temperature ( °C)	Treatment	Phenological Stage	No Damaged (%)	Only Pistil Damaged (%)	Only Stamen Damaged (%)	Pistil + Stamen Damaged (%)
−4	Control	Calyx red	43.0	39.5	17.5	0.0
	2% CNCs		85.9	1.3	12.8	0.0
	Control	First pink	1.8	28.9	38.0	31.3
	2% CNCs		29.7	0.0	70.3	0.0
−6	Control	Calyx red	0.0	55.5	1.8	42.7
	2% CNCs		9.8	57.0	6.2	27.0
	Control	First pink	0.0	6.0	0.0	94.0
	2% CNCs		1.4	22.8	2.4	73.4

^z^ The damage of flower buds was measured from the browning of each reproductive tissue.

## Data Availability

The data generated in this study are available from the corresponding author upon reasonable request.
